# Reduction in flippase activity contributes to surface presentation of phosphatidylserine in human senescent erythrocytes

**DOI:** 10.1111/jcmm.16010

**Published:** 2020-10-26

**Authors:** Momoko Seki, Nobuto Arashiki, Yuichi Takakuwa, Kosaku Nitta, Fumio Nakamura

**Affiliations:** ^1^ Department of Biochemistry School of Medicine Tokyo Women’s Medical University Tokyo Japan; ^2^ Department of Nephrology Tokyo Women’s Medical University Tokyo Japan

**Keywords:** ATP11C, flippase, microvesicles, phosphatidylserine, PLSCR1, scramblase, senescent erythrocytes

## Abstract

Mature human erythrocytes circulate in blood for approximately 120 days, and senescent erythrocytes are removed by splenic macrophages. During this process, the cell membranes of senescent erythrocytes express phosphatidylserine, which is recognized as a signal for phagocytosis by macrophages. However, the mechanisms underlying phosphatidylserine exposure in senescent erythrocytes remain unclear. To clarify these mechanisms, we isolated senescent erythrocytes using density gradient centrifugation and applied fluorescence‐labelled lipids to investigate the flippase and scramblase activities. Senescent erythrocytes showed a decrease in flippase activity but not scramblase activity. Intracellular ATP and K^+^, the known influential factors on flippase activity, were altered in senescent erythrocytes. Furthermore, quantification by immunoblotting showed that the main flippase molecule in erythrocytes, ATP11C, was partially lost in the senescent cells. Collectively, these results suggest that multiple factors, including altered intracellular substances and reduced ATP11C levels, contribute to decreased flippase activity in senescent erythrocytes in turn to, present phosphatidylserine on their cell membrane. The present study may enable the identification of novel therapeutic approaches for anaemic states, such as those in inflammatory diseases, rheumatoid arthritis, or renal anaemia, resulting from the abnormally shortened lifespan of erythrocytes.

## 1 INTRODUCTION

The lifespan of erythrocytes is approximately 120 days after generation and maturation in the bone marrow, and senescent erythrocytes are removed from circulation via phagocytosis by splenic macrophages. During this process, senescent erythrocytes are expected to expose phosphatidylserine (PS), which is usually localized asymmetrically in the inner leaflet of the cellular lipid bilayer,^1^, ^2^ although this is still debated.^3^ PS serves as an ‘eat me’ signal, which is recognized by macrophages, thereby initiating erythrocyte phagocytosis in macrophages.^2^, ^4^, ^5^ However, the mechanism underlying PS exposure on the surface of senescent erythrocytes remains poorly understood. Flippases, components of the type IV P‐type ATPase (P4‐ATPase) family, maintain PS localization in the inner leaflet by transporting PS from the outer leaflet of the cell membrane.^6^, ^7^ Conversely, several floppases, members of the ABC transporter family, utilize ATP and transport phospholipids non‐specifically from the inner leaflet of the cell membrane to the outer one.^6^ However, the PS transportation activity of floppases has been reported to be considerably lower than that of flippases in erythrocytes.^2^ ATP‐independent scramblases such as transmembrane protein 16F (TMEM16F), XK‐related protein 8 (Xkr‐8) and the phospholipid scramblase (PLSCR) family components facilitate the diffusion of phospholipids, including PS and phosphatidylcholine (PC), in random directions, causing the collapse of phospholipid asymmetry in the cell membrane.^8^, ^9^, ^10^ Therefore, we hypothesized that PS exposure on senescent erythrocytes occurs via reduction of flippase activity and/or promotion of scramblase activity. Although PS is transported from the outer to the inner leaflet by both flippase and scramblase, PS transportation rate is generally used to determine flippase activity. Thus, flippase activity should be calculated from the difference between the transportation rates of PS and PC.

Recently, we identified ATPase class VI type 11C (ATP11C) as an important flippase in human erythrocytes and demonstrated that point mutation in *ATP11C* resulted in ~90% reduction in PS transportation activity^11^ owing to improper membrane trafficking.^12^ We also reported that PLSCR1 serves as a scramblase in human erythrocytes and is suppressed by cholesterol under physiological conditions.^13^ Reduction in erythrocyte K^+^ concentration decreased flippase activity,^14^ and reduction in erythrocyte ATP levels decreased PS transportation rate.^15^, ^16^, ^17^ These factors are known to decrease in senescent erythrocytes.^18^, ^19^, ^20^, ^21^ Moreover, artificial elevation of erythrocyte Ca^2+^ concentration increased scramblase activity^9^, ^22^, ^23^, ^24^, ^25^ and/or decreased PS transportation from the outer to the inner leaflet in human erythrocytes.^26^ However, the significance of the effect of alteration in these factors on PS transportation in senescent erythrocytes remains unclear. Intracellular K^+^ concentration is regulated by four reported channels and transporters, including the Ca^2+^‐sensitive K^+^ channel termed Gardos channel (KCNN4); Na^+^ K^+^ ‐ATPase, K^+^/Cl^‐^ cotransporter and Na^+^/K^+^/2Cl^‐^ cotransporter.^27^, ^28^ Because intracellular K^+^ concentration depends on intracellular Ca^2+^ concentration by the Gardos channel, the influence of Ca*^2+^* and K^+^ concentration on PS transportation should be examined individually.

In this study, to clarify the mechanisms of PS exposure, we investigated the flippase and scramblase activities in senescent erythrocytes isolated using density gradient centrifugation and analysed the effect of intracellular concentrations of ATP, Ca^2+^ and K^+^ on lipid transportation activities in senescent erythrocytes. Moreover, we highlight the potential of ATP11C molecule, as a novel factor affecting flippase activity in senescent erythrocytes.

## 2 MATERIALS AND METHODS

### 2.1 Reagents

A23187 and valinomycin were purchased from Sigma‐Aldrich Inc (St. Louis, MO, USA); nitrobenzoxadiazole‐labelled PS (NBD‐PS) and NBD‐labelled PC (NBD‐PC) from Avanti Polar Lipids, Inc (Alabaster, AL, USA); dimethyl sulfoxide (DMSO) from Wako Pure Chemical (Osaka, Japan); bovine serum albumin (BSA) from Nacalai Tesque (Kyoto, Japan); rat IRDye 680RD and mouse IRDye 800 CW from LI‐COR Biosciences (Lincoln, NE, USA); and Percoll from GE healthcare (Chicago, IL, USA).

### 2.2 Preparation of washed erythrocytes and microvesicles and separation of senescent erythrocytes

This study was approved by the Ethics Committee of Tokyo Women's Medical University (#3835R). Written informed consent was obtained from all the study participants. Venous blood samples were collected from four healthy donors in blood collection tubes containing 1.5 mg/mL of EDTA‐2Na, and the samples were washed three times with Tris‐buffered saline (TBS; 150 mM NaCl, 25 mM Tris‐HCl, pH 7.4) for investigating Ca^2+^ clamped conditions or phosphate‐buffered saline (PBS; 137 mM NaCl, 2.68 mM KCl, 8.1 mM Na_2_HPO_4_, 1.47 mM KH_2_PO_4_, pH 7.4) for other experiments. Senescent erythrocytes were separated using density gradient centrifugation (33,500 g, 4°C, 15 min) using 89% Percoll in 10 mM Tris‐HCl, pH 7.4, 120 mM KCl and 0.25 M sucrose, as described previously.^29^ The senescent cells isolated from the lower layer were washed three times with PBS to remove residual Percoll. Erythrocytes isolated from the upper layer were used as controls.

Artificial microvesicles from the washed erythrocytes were prepared as follows. The cells were incubated in TBS, 20 mM Glucose, 2 mM CaCl_2_ and 5 µM A23187, a Ca^2+^ ionophore, for 30 min at 37°C to obtain an intracellular Ca^2+^ concentration of 2 mM. After gentle centrifugation at 15,000 g for 30 s, the resultant pellets and supernatants were collected separately; the pellets were washed twice with PBS and used as residual erythrocytes (R), while the supernatants were re‐centrifuged at 15,000 g for 30 min, and the resultant precipitates collected as microvesicles (Mv).^30^ The haemolysed residual erythrocytes and microvesicles were electrophoresed with haemolysed intact membrane (Int).

### 2.3 Measurement of flippase and scramblase activities

Washed erythrocytes at 5% of the haematocrit were pre‐incubated in 0.5 mL PBSG (PBS containing 20 mM glucose) at 37°C. Then, 0.5 µL NBD‐PS or NBD‐PC (1 mg/mL in DMSO) was added, and 20 µL of suspension was mixed with PBS containing 1% BSA at every 10 min until 20 min to remove the remaining NBD‐PS/PC in the outer leaflet. NBD‐PS/PC loading was determined by observing the fluorescence of the mixture treated without BSA using a flow cytometer (Cell Lab Quanta SC, BECKMAN COULTER, Brea, CA, USA). The proportion of PS/PC transported was calculated by dividing the fluorescence intensity of the inner leaflet by that of the loaded NBD‐PS/PC.^11^ The calculated fluorescent intensities were plotted, and the slope of the line was used as the NBD‐PS/PC transportation rate to calculate the flippase and scramblase activities.

### 2.4 Measurement of intracellular K^+^ and ATP concentration

Washed erythrocytes and senescent cells were haemolysed with the same volume of double‐distilled water and stored at −20°C. The intracellular K^+^ concentration was measured using the LAQUAtwin potassium ion meter (HORIBA, Kyoto, Japan). Intracellular ATP was measured using the ATP Assay Kit for blood (TOYO B‐Net Co., Tokyo, Japan) and a luminometer (Berthold LB 96V MicroLumat Plus; BERTHOLD, Bad Wildbad, Germany)　before and after removing haemoglobin by protein‐precipitating reagents, 0.6 M HClO_4_ and 2.5 M K_2_CO_3_.

### 2.5 Control of intracellular Ca^2+^ and K^+^ concentrations

To introduce Ca^2+^ into the erythrocytes, washed erythrocytes were incubated with 5 µM A23187 and Ca^2+^‐loading buffer [20 mM Tris‐HCl, pH 7.4, 1 mM EGTA, 100 mM KCl and 50 mM NaCl or 4.15 mM KCl and 145.85 mM NaCl, 20 mM glucose supplemented with various concentrations of Ca^2+^ (0‐0.3 µM)] at 37°C for 30 min.^13^ Intracellular free Ca^2+^ was determined by 1 mM EGTA, and various concentrations of CaCl_2_ were calculated by Calcon free software (eg 0.881 mM for final 0.3 µM). The cells were then washed with 1% BSA in Ca^2+^‐loading buffer without CaCl_2_ to remove A23187. The intracellular and extracellular Ca^2+^ concentrations were expected to be equal. BSA was removed by washing the cells three times with Ca^2+^‐loading buffer. K^+^ was introduced into erythrocytes using valinomycin, a potassium ionophore, in Tris buffer containing various concentrations of K^+^ (potassium introduction buffer; 2 µM valinomycin, 20 mM Tris‐HCl, pH 7.4, 1 mM EGTA, 20 mM glucose, 150 mM mixture of NaCl, and KCl) at 37°C for 30 min.^25^ The subsequent analyses were performed using the same potassium concentration buffer.

### 2.6 Measurement of mean corpuscular volume (MCV)

The MCV of control and senescent erythrocytes was measured using Sysmex pocH^®^‐80i analyzer (Sysmex, Kobe, Japan). The MCV of treated erythrocytes with artificially altered intracellular Ca^2+^ (under extracellular K^+^ of 4.15 mM) or K^+^ concentrations was also analysed. To resolve the inter‐individual differences in MCV, we converted the MCV to relative MCV (rMCV), which represents a percentage relative to the MCV of the treated erythrocytes at intracellular 0 µM Ca^2+^ or 100 mM K^+^.

### 2.7 Immunoblotting

Washed erythrocytes were haemolysed by mixing with 1 mM phenylmethylsulfonyl fluoride and 5P1E buffer (5 mM phosphate buffer, 1 mM EDTA) on ice and centrifuged at 20,400 g, 4°C, for 15 min. The pellets were washed three times with 5P1E buffer to remove the haemoglobin and obtain the erythrocyte membrane fraction. After ensuring equal protein concentrations by measuring the absorbance using the Bradford reagent (Thermo Fisher Scientific, Waltham, MA, USA) and the U‐5100 spectrophotometer (HITACHI, Tokyo, Japan), the erythrocyte membranes were mixed with the sample buffer, electrophoresed on 8% SDS‐polyacrylamide gel and transferred to a PVDF membrane. The PVDF membrane was blocked with 5% skim milk in TBS and incubated with the following primary monoclonal antibodies separately in T‐TBS (TBS and 0.1% Tween 20): rat anti‐ATP11C antibody (11C4; 1:1000^12^), mouse anti‐PLSCR1 antibody (1:5000; Abnova, Taipei, Taiwan) and mouse anti‐actin (1:50 000; Sigma‐Aldrich Inc). Thereafter, the membrane was washed three times and incubated with the following secondary antibodies in T‐TBS: rat IRDye 680RD for ATP11C and mouse IRDye 800 CW for PLSCR1 and actin. Fluorescence was detected and quantitated using a fluorescent scanner (Odyssey; LI‐COR). The levels of ATP11C and PLSCR1 were evaluated relative to the intensity of actin.

### 2.8 Analysis of PS exposure in senescent erythrocytes

One microlitre of packed control or senescent erythrocytes with different concentrations of calcium was suspended in 1 mL Annexin binding buffer (5 mM CaCl_2_ in TBS) and treated with 1 µL Annexin V‐FITC (MEBCYTO‐Apoptosis kit; MBL, Nagoya, Japan). The fluorescence levels of erythrocytes were quantified using flow cytometry. The erythrocytes with fluorescence values over 4000 were considered as erythrocytes with exposed PS.^11^

### 2.9 Statistical analysis

Data are expressed as means ± standard error. Statistical analyses were conducted by paired or unpaired two‐sided Student's t test or by one‐way ANOVA with Tukey‐Kramer HSD test using JMP software package, PRISM version 8.4.3 (GraphPad Software, La Jolla, CA, USA) or R v3.3.2.

## 3 RESULTS

### 3.1 Reduced flippase activity in senescent erythrocytes

**FIGURE 1:**
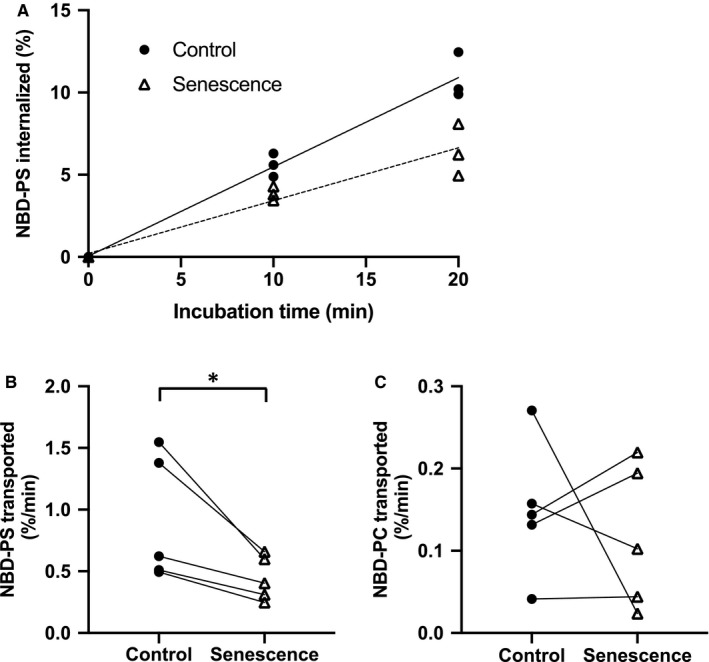
Flippase and scramblase activities in senescent erythrocytes. A, Proportion of NBD‐PS transported into the inner leaflet in control and senescent erythrocytes during 20 min. Both showed a liner increase up to 20 min (n = 3). B, Individual NBD‐PS transportation rates are shown (n = 5). *P < 0.05 vs control; paired t test. C, Individual NBD‐PC transportation rates show statistically no differences. (n = 5). NBD‐PS, nitrobenzoxadiazole‐labelled phosphatidylserine; NBD‐PC, nitrobenzoxadiazole‐labelled phosphatidylcholine

We first investigated whether decreased flippase activity and/or increased scramblase activity is involved in PS exposure in senescent erythrocytes. Here, we measured the transportation rates of NBD‐PS and NBD‐PC in control and senescent erythrocytes. PS transportation to the inner leaflet became linear during the 20‐min measurement period in all the erythrocytes (Figure 1A). NBD‐PS transportation rate in senescent erythrocytes was significantly lower (*P* < 0.05, n = 5) than that in the control erythrocytes (Figure 1B). However, NBD‐PC transportation rate in senescent erythrocytes was comparable to that in the control erythrocytes (Figure 1C). Therefore, the reduction of NBD‐PS transportation in senescent erythrocytes may not be the simple consequences of cell shrinkage in association with senescence.^31^ PS is transported by both flippase and scramblase, whereas PC is transported from the outer to the inner leaflet only by scramblase.^6^ Therefore, these results suggest that senescent erythrocytes show decreased flippase activity but not scramblase activity.

### 3.2 Reduction of intracellular ATP and K^+^ in senescent erythrocytes and decrease in flippase activity under low intracellular K^+^ concentrations

**FIGURE 2:**
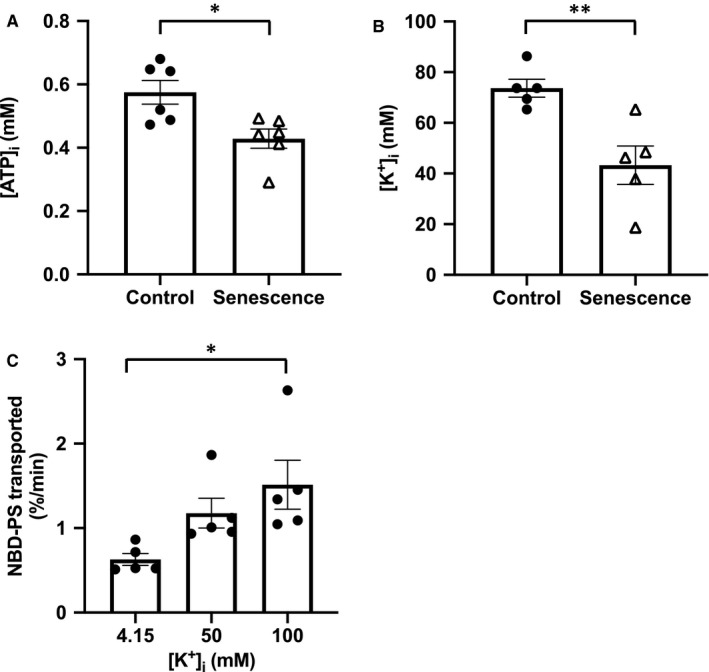
ATP and K^+^ concentrations in senescent erythrocytes and flippase activity under various intracellular K^+^ concentrations. A, Intracellular ATP levels in control and senescent erythrocytes. Control and senescent cells were haemolysed with the same volume of distilled water. ATP in the resultant supernatant was measured using the luciferase‐based luminescence assay (n = 6). *P < 0.05 vs control; unpaired t test. B, Intracellular K^+^ concentration in control and senescent erythrocytes (n = 5). **P < 0.01 vs control; unpaired t test. C, Transportation rates of NBD‐PS under various intracellular K^+^ concentrations (n = 5). *P < 0.05 vs cells with 100 mM intracellular K^+^; one‐way ANOVA with Turkey‐Kramer HSD test. NBD‐PS, nitrobenzoxadiazole‐labelled phosphatidylserine

Next, we assessed the ATP levels in senescent erythrocytes to investigate the effect of ATP reduction on flippase activity. ATP levels decreased from 0.57 ± 0.03 mM to 0.43 ± 0.03 mM in intracellular aqueous phase (*P* < 0.01, n = 6) for control and senescent erythrocytes, respectively (Figure 2A).

We also examined intracellular K^+^ concentration to assess the effect of K^+^ concentration on flippase activity in senescent erythrocytes. The intracellular K^+^ concentration in senescent erythrocytes was significantly lower than that in control erythrocytes (43 ± 7.6 mM vs 74 ± 3.5 mM in intracellular aqueous phase, respectively; *P* < 0.05, n = 5, Figure 2B). NBD‐PS/PC transportation rates were measured under various intracellular K^+^ clamped conditions. The intracellular K^+^ concentration was modulated using the specific potassium ionophore, valinomycin.^25^ PS transportation rates decreased with decreasing intracellular K^+^ concentration (Figure 2C), whereas PC transportation rates remained unaffected (not shown). At 43 mM K^+^, estimated intracellular K^+^ concentration of senescent erythrocytes (Figure 2B), flippase activity was approximately 30% lower than that at 100 mM K^+^.

### 3.3 Reduction of flippase activity under high intracellular Ca^2+^

**FIGURE 3:**
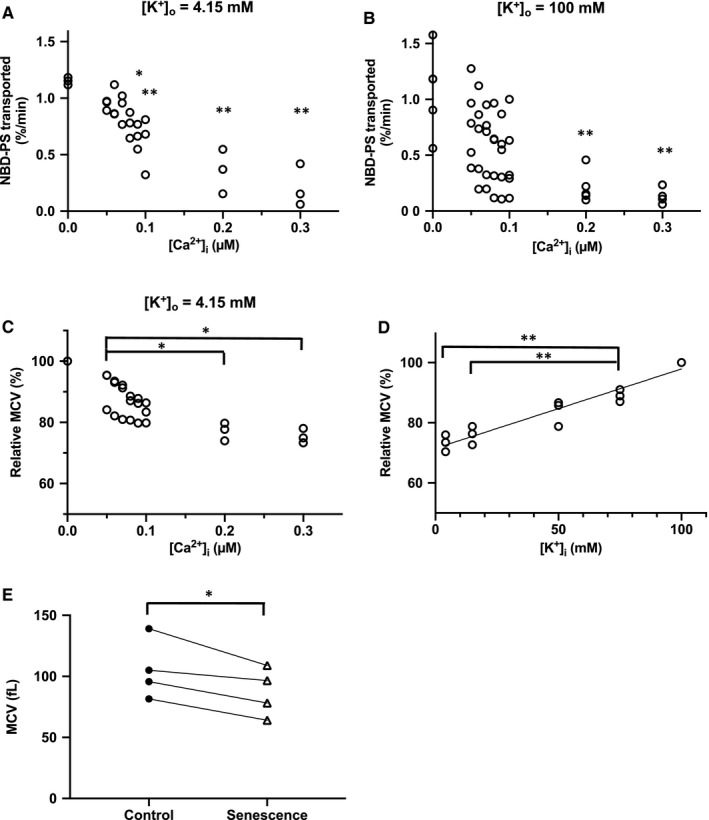
Flippase activity under various intracellular Ca^2+^ concentrations and estimation of intracellular Ca^2+^ and K^+^ concentrations using MCV. A and B, NBD‐PS transportation rate in erythrocytes with different intracellular Ca^2+^ concentrations in the presence of (A) extracellular 4.15 mM K^+^ (n = 3) and (B) extracellular 100 mM K^+^ (n = 5). *P < 0.05, **P < 0.01 vs cells with 0 µM intracellular Ca^2+^; one‐way ANOVA with Turkey‐Kramer HSD test. C and D, Relative MCV (rMCV) under various intracellular Ca^2+^ concentrations and K^+^ concentrations. rMCV values were calculated by considering MCV values at (C) intracellular 0 µM Ca^2+^ and (D) 100 mM K^+^ as 100 per cent, respectively (n = 3). *P < 0.05, **P < 0.01 vs rMCV under 0.05 µM intracellular Ca^2+^ concentration for (C) and 75 mM intracellular K^+^ concentration for (D); ANOVA with Turkey‐Kramer HSD test. E, MCV of control and senescent erythrocytes (n = 4). *P < 0.05 vs control; paired t test. MCV, mean corpuscular volume; rMCV, relative MCV; NBD‐PS, nitrobenzoxadiazole‐labelled phosphatidylserine

Next, to estimate the effect of intracellular Ca^2+^ on flippase activity, we measured PS transportation rates under modulated intracellular Ca^2+^ by utilizing the specific calcium ionophore, A23187 (Figure 3A).^26^ Intracellular K^+^ concentration is regulated by the Ca^2+^‐sensitive Gardos channel,^27^, ^28^ and intracellular K^+^ altered flippase activity (Figure 2C); therefore, we measured PS transportation rates under constant extracellular K^+^ concentration and varying intracellular Ca^2+^ concentration (Figure 3B). Increased intracellular Ca^2+^ concentrations reduced PS transportation rate, and this action was not affected by extracellular K^+^ concentration (Figure 3A and B). In contrast, PC transportation rate remained unchanged regardless of the increase in intracellular Ca^2+^ up to 0.3 µM (not shown). Thus, raising intracellular Ca^2+^ levels may inhibit flippase activity.

Due to our technical limitations, we could not measure erythrocyte intracellular Ca^2+^ concentration. Instead, we estimated the Ca^2+^ concentration in the senescent erythrocytes by comparing the MCV obtained from erythrocytes with artificially altered intracellular Ca^2+^ and K^+^ concentrations. Because of the high inter‐individual differences in MCV, we expressed relative MCV (rMCV) as a percentage relative to the MCV of erythrocytes at 0 µM Ca^2+^ and 100 mM K^+^. rMCV decreased with increasing intracellular Ca^2+^ concentration and decreasing K^+^ concentration (Figure 3C and D). The absolute MCV of senescent erythrocytes was 17.4% lower than that of the control erythrocytes (*P* < 0.05, n = 4, Figure 3E). Then, the intracellular Ca^2+^ concentration of senescent erythrocytes, that is the cells lessened 17.4%, was estimated to be approximately 0.1 µM from Figure 3C. The intracellular K^+^ concentration of senescent erythrocytes was also estimated to be 42 mM from Figure 3D. This value was good agreement with the measured K^+^ concentration in the senescent cells (Figure 2B).

### 3.4 Reduced expression of ATP11C in senescent erythrocytes

**FIGURE 4:**
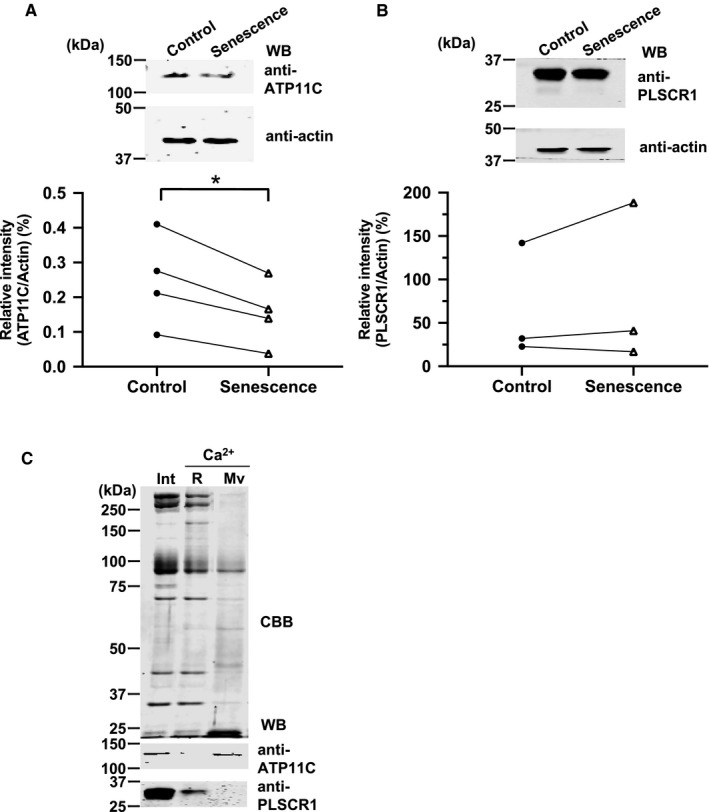
ATP11C and PLSCR1 expression in control and senescent erythrocytes and microvesicles. A, Upper: Representative Western blot images showing ATP11C and actin expression in control and senescent erythrocytes. Lower: Relative intensity of ATP11C in control and senescent erythrocytes (n = 4); the value was calculated using actin as the internal control. *P < 0.05 vs control; paired t test. B, Upper: Representative Western blot images for PLSCR1 and actin expression in control and senescent erythrocytes. Lower: Relative intensity of PLSCR1 in control and senescent erythrocytes (n = 3); the value was calculated using actin as the internal control. C, Upper: Representative images of CBB‐stained 8% acrylamide gels showing separation of membrane proteins prepared from intact erythrocytes (Int), residual erythrocytes (R) and microvesicles (Mv) produced artificially by increasing intracellular Ca^2+^ concentration to 2 mM. Lower: Representative Western blot images showing ATP11C and PLSCR1 signals. ATP11C, ATPase class VI type 11C; PLSCR1, phospholipid scramblase 1; Int, intact erythrocytes; R, residual erythrocytes; Mv, microvesicles

As a decrease in intracellular ATP and K^+^ cannot completely explain the reduction in flippase activity in senescent erythrocytes, we used Western blotting to investigate ATP11C expression on erythrocyte membrane. The ATP11C expression significantly decreased by 38% in the senescent erythrocytes, compared with that in control erythrocytes (*P* < 0.05, n = 4, Figure 4A). However, the expression of PLSCR1 (representing scramblase) remained unchanged between the senescent and control erythrocytes (Figure 4B).

Because previous reports suggested that microvesicles are released from ageing erythrocytes,^32^, ^33^, ^34^ we hypothesized that the reduction in ATP11C levels may reflect the loss of cell membrane via such microvesicles. Therefore, we assessed ATP11C levels using Western blotting in intact erythrocytes, residual erythrocytes and microvesicles isolated artificially by increasing intracellular Ca^2+^ to 2.0 mM. Immunoblot analysis using anti‐ATP11C antibody in intact erythrocyte membrane and microvesicles showed immunoreactive signals at 130 kDa. In contrast, PLSCR1 was not detected in microvesicles. Several proteins including ATP11C and PLSCR1 were reduced in the residual membranes, suggesting that they were digested by some proteases (Figure 4C). We also examined the immunoblot analyses of microvesicles separated from plasma. We found anti‐ATP11C immunoreactive signals in the microvesicles (not shown); however, we could not detect erythrocyte markers such as glycophorin A and band 3 in the same specimens. Thus, we concluded that the microvesicles in plasma may not be derived from senescent erythrocytes but from other tissues or organs.

### 3.5 Increased PS exposure in senescent erythrocytes

**FIGURE 5:**
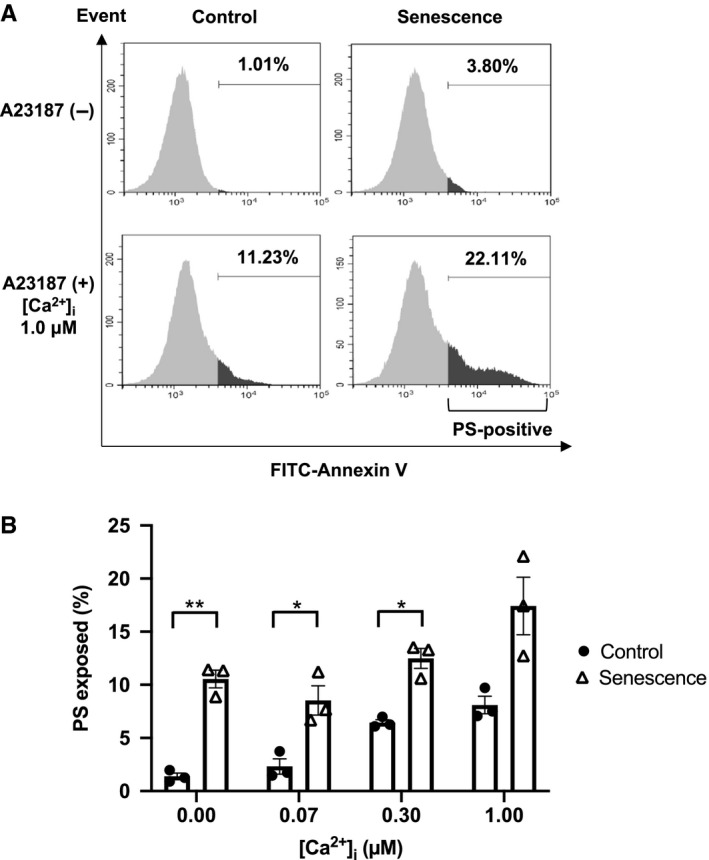
PS exposure in senescent erythrocytes. A, Annexin V‐FITC–based flow cytometry results showing PS exposure in control and senescent erythrocytes incubated without A23187 in EGTA buffer (upper) or A23187 in 1.0 µM Ca^2+^ loaded buffer (lower). B, Effect of different intracellular Ca^2+^ concentrations on PS exposure (n = 3). *P < 0.05, **P < 0.01 vs control; unpaired t test. PS, phosphatidylserine

In senescent erythrocytes, scramblase activity is expected to be more likely to exceed flippase activity, and PS exposure may occur at the end of the erythrocyte lifespan. To verify this, we compared the percentage of PS exposure in control and senescent erythrocytes and measured PS exposure when scramblase activity was increased by elevated intracellular Ca^2+^ concentration in erythrocytes. Further PS‐exposed cells were observed in senescent erythrocytes at each intracellular Ca^2+^ concentration except at 1.0 μM (*P* < 0.05, n = 3, Figure 5A and B).

## 4 DISCUSSION

In this study, we demonstrated that flippase activity decreased in senescent erythrocytes, while scramblase activity remained unchanged. Moreover, a decrease in intracellular K^+^, ATP and ATP11C reduced flippase activity, consequently contributing to PS exposure.

Consistent with previous reports, we found that ATP levels in senescent erythrocytes (0.43 ± 0.03 mM) were lower than those in control erythrocytes (0.57 ± 0.03 mM).^18^, ^19^, ^20^ Flippase activity, calculated using concentrations based on previously reported Michaelis constant (K_m_) for ATP (0.23 mM) in erythrocytes,^35^ was estimated to be lower by ~9% in senescent erythrocytes than in control erythrocytes. We also demonstrated that decreased K^+^ concentration in senescent erythrocytes contributes to reducing flippase activity by ~30%. Thus, reduction of intracellular ATP and K^+^ may collectively decrease flippase activity in senescent erythrocytes. However, the flippase activity in senescent erythrocytes decreased by approximately 50‒70% from control erythrocytes (Figure 1B). This reduction could not be fully explained by the decreased intracellular ATP and K^+^ concentrations in senescent erythrocytes, suggesting the involvement of an additional factor in flippase activity reduction.

We assessed the amount of the flippase ATP11C, which accounts for 90% of the flippase activity in erythrocytes.^11^ We found a 38% lower ATP11C level in senescent erythrocytes. It has been shown that microvesicles are released from ageing erythrocytes;^32^, ^33^, ^34^ then, ATP11C might be removed from the erythrocyte membranes and transferred to the microvesicles. We have demonstrated that ATP11C was removed from erythrocytes via artificially generated microvesicles. In addition, we could detect ATP11C in plasma‐derived microvesicles but not erythrocyte markers in the same specimens (not shown). Thus, this ‘removal by microvesicle’ model is poorly proven in vivo. Another possible reason for this reduction is that ATP11C in senescent erythrocytes would be cleaved by calpain^36^ or caspase 3.^37^ This could also explain that reduction of ATP11C in the Ca^2+^‐treated residual membranes as shown in Figure 4C.

Recently, we identified that ATP11C T418N mutation results in mild haemolytic anaemia due to improper membrane trafficking.^12^ The percentage of PS‐positive cells in senescent erythrocytes of patients was more than that in normal senescent erythrocytes;^11^ therefore, the residual flippase activity in normal senescent erythrocytes contributes to maintaining PS in the inner leaflet and achieving the 120‐day lifespan. Furthermore, PLSCR1 was undetectable in microvesicles, and increase in scramblase activity induced by elevation of intracellular Ca^2+^ caused increased PS exposure in both control and senescent erythrocytes, suggesting that PLSCR1 levels do not decrease in senescent erythrocytes. The reason for the presence of ATP11C and absence of PLSCR1 in microvesicles may be the difference in their association with the cytoskeleton; PLSCR1 may be tightly bound to the cytoskeleton, which is absent in microvesicles.

We attempted but failed to measure intracellular Ca^2+^ concentration using fluorescent indicators, probably due to our technical limitations in dealing with a high level of haemoglobin as a pigment interfering the measurement. Another possible reason is that the reported erythrocyte Ca^2+^ concentration is 30‒60 nM,^38^ and thus, the difference between control and senescent erythrocytes may be too subtle to measure. Therefore, we estimated intracellular Ca^2+^ concentration from the alteration of MCV and assumed that intracellular Ca^2+^ is approximately 100 nM in senescent erythrocytes (Figure 3C). Based on these concentrations, flippase activities in control erythrocytes and senescent erythrocytes were estimated to be 0.9%/min and 0.6%/min, respectively (Figure 3A). Thus, alteration of intracellular Ca^2+^ may reduce flippase activity by 33% in senescent erythrocytes. As the intracellular K^+^ was decreased in senescent erythrocytes (Figure 2B), the activation of K^+^ channels and transporters such as Ca^2+^ sensitive K^+^ channel (Galdos channel, KCNN4), Na^+^/K^+^‐ATPase, K^+^/Cl^‐^ cotransporter and Na^+^/K^+^/2Cl^‐^ cotransporter were expected.^27^, ^28^ However, as the EC50 of Gardos channel for intracellular Ca^2+^ is estimated to be 4.7 µM,^39^ 100 nM Ca^2+^ in senescent erythrocytes would not activate the channel. As the K_m_ of Na^+^/K^+^‐ATPase for ATP in rat is reported to be 0.59 mM,^40^ reduced ATP in senescent erythrocytes would prevent incorporation of K^+^, resulting in the reduction of K^+^. The K^+^/Cl^‐^ cotransporter is reported to be involved in K^+^ leakage in young erythrocytes.^41^ Na^+^/K^+^/2Cl^‐^ cotransporter is reported to regulate steady‐state volume of erythrocytes^42^; therefore, the reduction of K^+^ concentration in senescent erythrocytes might reflect other mechanisms such as the decreased activity of Na^+^/K^+^‐ATPase and/or Na^+^/K^+^/2Cl^‐^ cotransporter.

**FIGURE 6:**
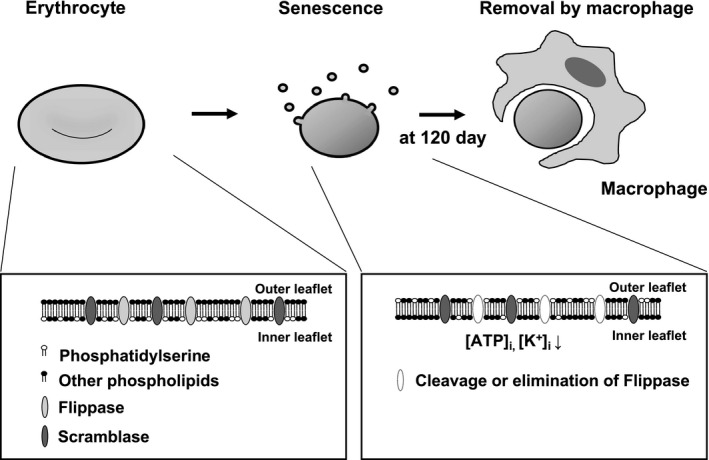
Erythrocyte lifespan and mechanisms underlying PS exposure in senescent erythrocytes. Around 120 days after their maturation, erythrocytes expose PS in the outer leaflet of their cell membrane. Macrophages detect such senescent erythrocytes via the exposed PS and phagocytose them, thus removing them from circulation. Senescent erythrocytes show significantly decreased flippase activity. Multiple factors, including intracellular ATP and K^+^ concentration and reduced ATP11C expression, contribute to the decrease in flippase activity, thereby maintaining PS exposure in senescent erythrocytes. PS, phosphatidylserine; ATP11C, ATPase class VI type 11C

In conclusion, the present study revealed that decreased intracellular ATP and K^+^ levels and ATP11C expression reduces flippase activity in senescent erythrocytes (Figure 6). Furthermore, our findings suggest that elevation of intracellular Ca^2+^ also reduces flippase activity. These factors may be affected in disorders involving an abnormally shortened lifespan of erythrocytes, such as inflammatory diseases,^43^ rheumatoid arthritis^44^ or renal anaemia.^45^ Further studies focused on alteration of flippase activity in these diseases may enable the identification of potential therapeutic targets and development of novel treatment approaches in anaemic states for such diseases.

## CONFLICT OF INTEREST

The authors declare no conflicts of interest.

## AUTHOR CONTRIBUTIONS

Momoko Seki: Conceptualization (equal); Data curation (lead); Formal analysis (equal); Funding acquisition (equal); Investigation (lead); Resources (equal); Validation (equal); Visualization (equal); Writing‐original draft (lead). Nobuto Arashiki: Conceptualization (lead); Formal analysis (equal); Funding acquisition (equal); Investigation (equal); Methodology (lead); Project administration (lead); Writing‐review & editing (equal). Yuichi Takakuwa: Conceptualization (equal); Formal analysis (supporting); Funding acquisition (equal). Kosaku Nitta: Supervision (equal); Writing‐original draft (equal). Fumio Nakamura: Conceptualization (equal); Formal analysis (lead); Funding acquisition (equal); Methodology (supporting); Project administration (equal); Supervision (lead); Writing‐review & editing (lead).
